# Genetic Variation and Biological Control of *Fusarium graminearum* Isolated from Wheat in Assiut-Egypt

**DOI:** 10.5423/PPJ.OA.09.2015.0201

**Published:** 2016-04-01

**Authors:** Amer F. Mahmoud

**Affiliations:** Department of Plant Pathology, Faculty of Agriculture, Assiut University, 71526 Assiut, Egypt

**Keywords:** *Bacillus subtilis*, biocontrol, FHB, *Fusarium graminearum*, SRAP, *Trichoderma harzianum*

## Abstract

*Fusarium graminearum* Schwabe causes Fusarium head blight (FHB), a devastating disease that leads to extensive yield and quality loss of wheat and other cereal crops. Twelve isolates of *F. graminearum* were collected from naturally infected spikes of wheat from Assiut Egypt. These isolates were compared using SRAP. The results indicated distinct genetic groups exist within *F. graminearum*, and demonstrated that these groups have different biological properties, especially with respect to their pathogenicity on wheat. There were biologically significant differences between the groups; with group (B) isolates being more aggressive towards wheat than groups (A) and (C). Furthermore, *Trichoderma harzianum* (Rifai) and *Bacillus subtilis* (Ehrenberg) which isolated from wheat kernels were screened for antagonistic activity against *F. graminearum.* They significantly reduced the growth of *F. graminearum* colonies in culture. In order to gain insight into biological control effect *in situ*, highly antagonistic isolates of *T. harzianum* and *B. subtilis* were selected, based on their *in vitro* effectiveness, for greenhouse test. It was revealed that *T. harzianum* and *B. subtilis* significantly reduced FHB severity. The obtained results indicated that *T. harzianum* and *B. subtilis* are very effective biocontrol agents that offer potential benefit in FHB and should be harnessed for further biocontrol applications. The accurate analysis of genetic variation and studies of population structures have significant implications for understanding the genetic traits and disease control programs in wheat. This is the first known report of the distribution and genetic variation of *F. graminearum* on wheat spikes in Assiut Egypt.

Fusarium head blight (FHB), caused by the fungal plant pathogen *Fusarium graminearum*, is an important disease in cereal crops causing major economic losses of 20–100% (McMullen et al., 1997 and Manning et al., 2000). Diseased spikelets exhibit symptoms of premature bleaching shortly after infection. The fungus produces a mycotoxin that poses a significant threat to the health of domestic animals and humans. The major toxin produced by *F. graminearum* in association with FHB in wheat and barley is deoxynivalenol (Pestka and Smolinski, 2005 and McMullen et al., 1997). Mycotoxins produced by *Fusarium species* result in a loss of yield and reduced quality of grains. Fusarium toxins including the trichothecenes nivalenol (NIV), deoxynivalenol (DON) and its derivatives 3- and 15-acetyldeoxynivalenol (3-ADON, 15-ADON) contaminate cereal products and have been shown to be harmful to humans, animals, and plants (Desjardins and Hohn, 1997; Desjardins et al., 1993 and Goswami and Kistler, 2004). Sequence related amplified polymorphism (SRAP) technology has been recognized as one of the most variable types of DNA sequences found in plants. This SRAP system has been employed for mapping and gene tagging in *Brassica* (Li and Quiros, 2001). SRAP marker is homogenously distributed in the genome and could produce higher polymorphism than those from AFLP, RAPD, and SSR markers. It has been employed to evaluate genetic diversity and phonetic relationships among *Turfgrass species* (Budak et al., 2004a). The polymorphism produced by SRAP (95%) marker technique was higher than those produced by ISSR (81%), RAPD (79%), and SSR (87%) (Budak et al., 2004b). The SRAP marker technique was used as a new technique to assess genetic relationships and diversity among genotypes of *Saccharum*. The level of observed polymorphism proved that the SRAP system was robust at amplifying markers across species and genera and did so according to the evolutionary history interconnecting members of the Saccharum complex (Suman et al., 2008). Cloning and sequencing of a set of cDNA to visualize transcript polymorphism are reported using SRAP technology in three *Bentgrass species*. The ESTs identified in this study could potentially be used in Turfgrass breeding and genetics programs as functional markers. Integration of these ESTs to the existing linkage map of *Turfgrass species* provides high-density coverage in selected genomic regions. Minimum evolutionary tree clustering indicated that ESTs obtained using SRAP could be used for comparative genomics analysis of transcribed genes among the grass species (Dinler and Budak, 2008). Furthermore, Baysal et al., 2009 use SRAP primers to study the population and genetic relationships within and among *Fusarium oxysporum* f. sp. *lycopersic*i races. Mutlu et al., 2008 reported the tagging of the gene for resistance to Fusarium wilt (FOM) in eggplant using SRAP, RGA, SRAP-RGA and RAPD markers. Molecular markers are useful tools in the analysis of genetic variation in populations of plant-pathogenic fungi. A number of molecular techniques are available for studying the genetic relationships within and among fungal populations within a species. Sixty isolates of *F. graminearum*, the causal pathogen of Fusarium head blight, were compared using vegetative compatibility analysis and Polymerase Chain Reaction (PCR)-based Sequence Related Amplified Polymorphisms (SRAP) (Fernando et al., 2006). SRAP is based on two-primer amplification to amplify the ORFs. In a gene, ORFs are located between the start-code sequence (initiation codon) and the stop-code sequence (termination codon) (Li and Quiros, 2001). The analysis of sequenced SRAP fragments targets into hypothetical proteins from different *Fusarium species* showing that the SRAP technique not only allows studying *F. poae* genetic variability, but also targets coding regions into the *F. poae* genome. Genetic variability of *F. poae* using SRAP technique also demonstrates the efficacy of this molecular marker to amplify open reading frames in fungus (Dinolfo, et al., 2015). In this study SRAP analysis was used to determine the genetic variation of *F. graminearum* isolates.

Biological control of *F. graminearum* has shown promise in previous studies due to their low environmental impact, and their ability to help reduce growers’ dependency on chemicals, thereby slowing the development of fungicide resistance in pathogen populations (Crane et al., 2013; Jochum et al., 2006). Several bacteria or fungal strains have been reported to have antagonistic effects against *F. graminearum* (Hue et al., 2009). *Trichoderma species* are biological control agents that control ascomycetous and basidiomycetous fungi, which are mainly soil-borne but also airborne pathogens. Antagonists of phytopathogenic fungi have been used to control plant diseases, and 90% of such applications have been carried out with different strains of the fungus Trichoderma (Monte, 2001). The genus Trichoderma comprises a great number of fungal strains that act as biological control agents, the antagonistic properties of which are based on the activation of multiple mechanisms. Trichoderma can indirectly biocontrol phytopathogens by competing for nutrients and space nutrients, through the secretion of antibiotic volatiles and/or diffusible metabolites, which modify soil conditions promoting growth and plant defense mechanisms. Moreover, mycoparasitism is considered a direct biocontrol mechanism (Benítez et al., 2004; Howell, 2003). The addition of Trichoderma metabolites that may act as elicitors of plant resistance, or the expression in transgenic plants of genes whose products act as elicitors, also results in the synthesis of phytoalexins, PR proteins and other compounds, and in an increase in resistance against several plant pathogens, including fungi and bacteria (Dana et al., 2001; Elad et al., 2000). Bacterial isolates obtained from rhizosphere and kernel of wheat was reported for control Fusarium head blight (Stockwell et al*.*, 1999). Among them, *Bacillus* strains are well-known antibiotic producers, which have advantage over other biocontrol microorganisms due to their inherent property to form endospores and resistance to extreme conditions. The antagonistic effects of *Bacillus* strains have been shown by *in vitro* antibiosis (Chan et al., 2003) and *in situ* disruption of spikelet infection leading to reduced disease severities (Khan et al., 2001). *B. subtilis* was demonstrated to be the most effective in reduction, affecting fungal growth parameters and toxin production *in vitro* at all the tested incubation periods (Cavaglieri et al., 2005). *Bacillus species*, as a group, offer several advantages over other bacteria for protection against root pathogens because of their ability to form endospores and the broad-spectrum activity of their antibiotics. There are numerous reports of *Bacillus species* which repress pathogens (Bacon et al., 2001; Estévez de Jensen et al., 2002). Therefore, this study was carried out with the objective of evaluating the efficacy of *Trichoderma harzianum* and *Bacillus subtilis* in management of FHB caused by *F. graminearum.*

## Materials and Methods

### Isolation and identification of *Fusarium graminearum.*

A total of fifteen wheat fields in three zones of Assiut governorate were sampled during March and April 2011. Ear bleaching and spikelet bleaching with FHB symptoms were collected. The infected wheat heads were cut into 0.5 cm long pieces. These were surface sterilized in 3% sodium hypochlorite solution, rinsed twice in sterile distilled water and blot dried between sterile filter paper. The surface sterilized pieces were placed onto potato dextrose agar (PDA) amended with streptomycin sulphate (120 mg/l) and incubated for five days at 25°C. Fungal colonies were identified based on cultural and morphological characteristics like mycelial colour, pigmentation, spore shape, septation and sporophores. All colonies with characteristic growth patterns of FHB pathogens were transferred on to fresh PDA; those with growth patterns typical of *Fusarium species* were also plated onto Sucrose Nutrient Agar (SNA) (Nirenberg, 1981). Plates were incubated at 25°C under 12 hrs day light and 12 hrs darkness cycles for 10 days and cultures identified to species level based on colony characteristics on PDA and spore morphology on SNA according to Nelson et al., 1983. Hyphae were stained with 0.05% trypan blue in lacto-phenol (Meyer et al., 1998) and examined under a compound microscope to determine hyphal morphology. All isolates of *F. graminearum* were purified by single spore isolation.

### Pathogenicity tests

Twelve isolates of *F. graminearum* were tested for the ability to infect wheat plants in greenhouse of Plant Pathology Department, Faculty of Agriculture, Assiut University in October 2011. A medium susceptible Egyptian wheat cultivar (Sakha-69) was used for the investigation. Wheat seeds were planted in sterilized pots (25 × 25 cm^2^) containing a peat/sand mixture (ten seeds per pot) with four pots per isolate (replicates). The pots were kept moist and moved to random positions on the greenhouse bench following a complete randomized design, and grown at 25 ± 2°C during day and 17 ± 2°C during night. *F. graminearum* conidial inoculums was prepared with Mung Bean Agar Medium (MBA) (Bai and Shaner, 1996). Each isolate was cultured separately at 25 ± 1°C for14 days and used to inoculate wheat plants.

Conidia suspension of each isolate was harvested and adjusted to 5 × 10^5^ conidia/ml. Three drops (0.01%) of Tween 20 was added to ensure uniform conidia dispersion. Wheat spikes were inoculated at 50% flowering (GS65, Zadoks et al., 1974) by spraying with hand sprayer, exposing all spikelets to the inoculum. Controls were treated similarly with distilled water only. After inoculation, the spikes were incubated under polythene bags for 48 hrs to ensure high relative humidity for optimal infection. Each isolate was inoculated separately and replicated three times during 10 days.

### Disease assessment

Fusarium head blight was assessed as a percentage of heads showing disease symptoms, on ten average sized spikes per replicate. The number of infected spikelets/head was recorded at two dates (14 and 28 days after inoculation) and adjusted to the total number of spikelets/heads. The relative number of infected spikelets of the two assessment dates was averaged (Cumagun and Miedaner, 2003; Mesterhazy, 2002; Snijders and Perkowski, 1990).

### Fungal cultivation for DNA extraction

Isolates were cultured on potato dextrose agar (PDA) for 7 days at 25°C. Mycelia of each isolate were prepared in a flask (250 ml) with 100 ml of Potato Dextrose Broth (PDB). To obtain mycelia, PDB flasks were inoculated with a 0.5 ml suspension of approximately10^5^ conidial spores per milliliter of an isolate. The flasks were incubated at 25°C for 7 days without agitation. The mycelia were harvested by filtration through two layers of sterilized miracloth, frozen with liquid nitrogen and stored at −80°C until lyophilized. Before proceeding with nucleic acid extraction, the mycelium was ground in liquid nitrogen in a sterile mortar to obtain a mycelium powder.

### DNA extraction

Total genomic DNA was extracted from lyophilized mycelium according to the CTAB (hexacetyltrimethylammonium bromide; Sigma-Aldrich) a modification of miniprep protocol described by O’Donnell et al., 1997 and Voigt et al., 1999 was used. Approximately 50 mg of pulverized mycelium was re-suspended in 700 μl of CTAB extraction buffer (100 mM Tris-HCl [pH 8.4], 1.4 M NaCl, 25 mM EDTA, 2% CTAB) and vortexed for 10 seconds. Following extraction, an equal volume of chloroform was added to each tube, vortexed for 5 seconds, and then spun for 10 minutes at 12,300 × g in a microcentrifuge (Eppendorf AG-Centrifuges 5415D). A 500 μl portion of the upper phase was removed to a new 1.5 ml tube, and DNA was precipitated by the addition of an equal volume of −20°C isopropanol. After the DNA was pelleted at 12,300 × g in a microcentrifuge for 1 min, the supernatant was discarded and resulting pellets were washed twice with 70% ethanol. Pellets were each air-dried and re-suspended in 100 μl of TE buffer (10 mM Tris-HCl [pH 8.0], 1 mM EDTA [pH 8.0]). RNA contamination was removed by incubating each preparation with 40 μg/ml RNase A (Sigma) at 37°C for 30 min. The concentration of DNA was determined by spectrophotometry with a nano-drop spectrophotometer ND-1000 (NanoDrop Technologies) at A_260_.

### SRAP analysis

The SRAP analysis was carried out using 16 primer combinations (Table 1). The PCR reaction was set up in a final volume of 20 μl containing 50 ng of template DNA, 1× PCR buffer, 1.5 mM MgCl_2_, 0.2 mM dNTPs mix, 0.1 mM forward primer, 0.1 mM reverse primer and 1 unit of Taq polymerase; final volume completed to 20 μl with sterile ddH_2_O (Li and Quiros, 2001 and Budak et al., 2004a with minor modifications). Amplifications were performed in mastercycler gradient-thermal cycler (Eppendorf, Germany) programmed for 5 min at 95°C for initial denaturation (one cycle); followed by 35 cycles of 1 min at 94°C for denaturation, 1 min at 47°C for annealing, 1 min at 72°C for extension, and ending with 5 min at 72°C for a final extension (one cycle) (Buddak et al., 2004c). Amplified DNA was analyzed by electrophoresis in 1.0% agarose gel, then stained with ethidium bromide (0.5 μg/ml) and observed under UV light in gel documentation system (Bio-Rad).

### Analysis of SRAP-PCR data

Digital images were scored as ‘1’ for presence and ‘0’ for absence of clear and unambiguous DNA fragments. Similarity matrix was constructed from the binary data with Jaccard’s coefficients (Jaccard, 1908). The genetic distance matrix was subjected to cluster analysis using the Unweighted Pair-Group Method with Arithmetic mean (UPGMA) in NTSYS-pc version 2.1 program (Rohlf, 2000).

### Isolation and identification of antagonists

*Trichoderma harzianum* and *Bacillus subtilis* were isolated from wheat kernels. *T. harzianum* was isolated on PDA medium at 26°C and identified based on morphological characteristics of mycelia and conidiophores as described by Domsch et al*.*, 1980 and Dhingra and Sinclair, 1995. Whereas, *B. subtilis* was isolated on Nutrient Agar (NA) at 28°C, and identified based on morphological, culture and biochemical activities according to Skinner and Lovelock, 1979 and Sneath et al., 1986.

### Efficacy of *T. harzianum* and *B. subtilis* against *F. graminearum in vitro*

*Antagonistic capability of seven isolates of T. harzianum* and five isolates of *B. subtilis* were tested against the highly pathogenic isolate (*F.g*.8) of *F. graminearum in vitro.* Dual culture technique was followed; mycelial disks 5 mm in diameter were cut from the edges of actively growing colonies of *F. graminearum* and Trichoderma isolates, and were placed opposite each other, 1.5 cm from the edge of 9 cm Petri dishes containing PDA. Petri dishes inoculated with *F. graminearum* alone served as controls. Each pair was replicated four times and incubated for four days at 25°C in darkness, then scored for degree of antagonism using the 1–5 scale of Bell et al. (1982) and Mahmoud and Abo-Elyousr (2014): 1, Trichoderma completely overgrew the pathogen and covered the entire Petri dish; 2, Trichoderma overgrew at least two thirds of the Petri dish; 3, Trichoderma and *F. graminearum* each colonized 50% of the medium surface and neither organism appeared to dominate the other; 4, *F. graminearum* colonized at least two-thirds of the medium surface and appeared to withstand the encroachment of Trichoderma; 5, *F. graminearum* completely overgrew the entire Petri dish. An isolate of Trichoderma was considered to be antagonistic to the pathogen if the mean score for a given comparison was ≤2, but not highly antagonistic if the score was ≥3. For test the efficacy of *B. subtilis*, the pathogen agar disc was inoculated at the middle of plate and the antagonist at two equidistant points located 1.5 cm from plate edge. Degree of antagonism was determined by measuring the pathogen colony diameters and percentage inhibition calculated:

$$\text{Inhibition }(\%)=\frac{\text{A}-\text{B}}{\text{A}}\times 100$$

Where: (A) is the colony diameter of pathogen alone (control); (B) is the colony diameter of pathogen after antagonist effect.

### Efficacy of *T. harzianum* and *B. subtilis* on controlling FHB under greenhouse conditions

Ten seeds of susceptible wheat cultivar (Sakha-69) were sown, and the inoculum of the highly pathogenic isolate (*F.g*.8) of *F. graminearum* was produced in MBA (Bai and Shaner, 1996) as described previously in pathogenicity tests. Conidia suspension of *F. graminearum* was harvested and adjusted to 5 × 10^5^ conidia/ml. Three drops (0.01%) of tween 20 was added to ensure uniform conidia dispersion. *T. harzianum* isolate (*T. h*.3) and *B. subtilis* isolate (*B. s.*2) with great inhibition zone *in vitro* against *F. graminearum* were investigated for their ability to reduce the incidence of head blight in wheat. Inoculum of *T. harzianum* was prepared using PDB for 14 days at 26°C. Inoculums was harvested by passing the liquid culture through double layer cheesecloth, and adjusted to 5 × 10^5^ spore/ml. While *B. subtilis* was grown on Nutrient Broth medium for 48 hrs at 28°C and inoculums was adjusted to 2 × 10^5^ cfu/ml with fresh medium. Wheat spikes were inoculated at 50% flowering (GS65, Zadoks et al., 1974) by spraying with hand sprayer, exposing all spikelets to the inoculum. Inoculation with *F. graminearum* began at 5 hr after the inoculation with *T. harzianum* and *B. subtilis*. Positive controls were inoculated similarly with *F. graminearum* only, while negative controls were treated with distilled water. There were four replicate pots per treatment. Pots were arranged in complete randomized design. After inoculation, the spikes were incubated under polythene bags for 48 hrs to ensure high relative humidity for optimal infection. Each isolate was inoculated separately and replicated three times during 10 days. Head blight was evaluated as described previously in pathogenicity tests.

### Statistical analysis

The results were analyzed using ANOVA test and the means differences were regarded as significant using LSD test at 5% level of probability according to Gomez and Gomez, 1984.

## Results

### Collection and identification of *F. graminearum* isolates

Wheat fields were surveyed to access the incidence of FHB during wheat growing season, from March to the beginning of May, in 2011. Fifteen fields representing three districts of Assiut governorate namely Dirout, Manfalout and Abuteeg were surveyed. The geographic origins of the isolates collected are given in Table 2. The results indicated that, the morphological characteristics of twelve isolates were found to be identical to those of *F. graminearum*. Isolates were identified as *F. graminearum* on the basis of growth rate, pigmentation of colonies on PDA, spore morphology on SNA as well as morphology and size of microconidia and macroconidia according to Nelson et al. (1983) and Summerell et al. (2003). Hyphae were stained with 0.05% trypan blue in lacto-phenol (Meyer et al., 1998) and examined under a compound microscope to determine hyphal morphology.

### Pathogenicity tests

FHB was assessed at two dates (14 and 28 days) after inoculation. All *F. graminearum* isolates caused visible head blight symptoms under greenhouse conditions. No symptoms of disease occurred in uninfected control. The wheat cultivar Sakha-69 was extremely susceptible to all of the isolates tested under greenhouse conditions. Means of FHB severity ranged from 54.25 to 92.50 %, averaging 68% in total. There were significant differences in disease severity among the twelve *F. graminearum* isolates. These differences were observed on the two dates on which the percentage of diseased spikelets was calculated. The percentage of diseased spikelets increased with time for the twelve *F. graminearum* isolates (Table 2). Based on the obtained results, the most aggressive isolates were *F.g.*8 followed by *F.g.*6 and *F.g.*9 which isolated from Manfalout. Whereas, the least aggressive isolates were *F.g.*11 and *F.g.*12, which isolated from Abuteeg. On the other hand, results show that, *F. graminearum* isolates obtained from Dirout were varied significantly, as some of these isolates associated with the low level of disease severity *F.g.*2 (62.75%) and some have a high level of aggressiveness such as *F.g.*5 (79.50%). The results also, revealed that, the percentage of diseased spikelets differed significantly among isolates of Manfalout. The most aggressive isolate showing the highest disease severity was the isolate *F.g.*8 (92.50%), while the least aggressive one was the isolate *F.g.*7 (78.25%). Therefore, isolate *F.g.*8 was selected for further work in biological control. On the other hand, the results showed that, *F. graminearum* group (B) is more pathogenic to wheat than groups (A) and (C), although the pathogenicity of individual isolates within each group was varied. The virulent of group (B) is potentially due to the differences in Fusarium mycotoxins production. Mycotoxins, predominantly are trichotecenes i.e nivalenol (NIV) and deoxynivalenol (DON). Previous publications confirmed that producing mycotoxins; such as trichothecenes, zearalenone and fumonisins, are associated with the most aggressive isolates. DON is necessary to suppress plant defense enabling the pathogen to break through the rachis node. DON production is strongly induced, most likely by the host, at this point of infection (Carter et al., 2002; Ilgen et al., 2009; Jansen et al., 2005). Moreover, in wheat and maize, trichothecene biosynthesis alters strain aggressiveness (Proctor et al., 1995), with DON-producing strains being perceived as more virulent than NIV-producing strains (Desjardins et al., 2008). Also, this observation was supported by results obtained by Kimura et al. (1998) and Alexander et al. (1998).

### SRAP analysis

**Fig. 1:**
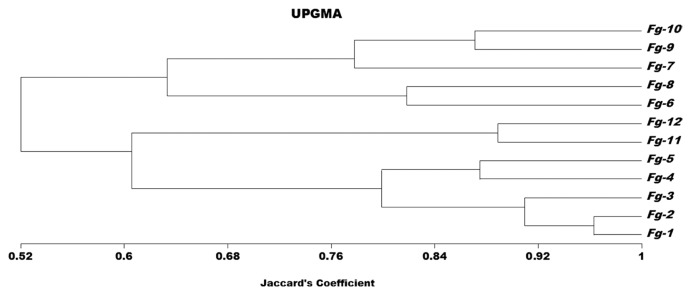
Dendogram showing genetic relatedness among *F. graminearum* isolates based on SRAP analysis.

Genetic variation was detected among twelve isolates of *F. graminearum* using Sequence Related Amplified Polymorphism (SRAP) and it has been indicated a wide variation among all isolates of *F. graminearum*. Among the sixteen SRAP primer combinations, seven were amplified the genomic DNA of *F. graminearum*, and produced 2–8 bands ranging from 75–5,000 bp. The number of amplified DNA fragments varied, depending upon the primers and isolates used. The primers Em2- Me6, Em3- Me4, Em3- Me10, Em6- Me4, Em11- Me4, Em11- Me6 and Em14- Me3 amplified the genomic DNA of all isolates of *F. graminearum* and producing fingerprint profiles, which were clearly distinguished among the different isolates of *F. graminearum*. A dendrogram constructed using the SRAP data (Fig. 1) shows the isolates to be divided into three groups. The tested isolates were clustered together according to the geographic areas. The isolates of *F. graminearum* obtained from Dirout, Manfalout and Abuteeg were clustered together with a genetic similarity of 52%. Isolates obtained from Dirout (group A), were clustered together with a genetic similarity of 79%. While, isolates obtained from Manfalout (group B), were clustered together with a genetic similarity of 63%. Isolates *F.g*.11 and *F.g*.12 obtained from Abuteeg (group C) were clustered together and displayed high genetic similarity of 88%. The analysis also indicated that isolates: *F.g*.4, *F.g*.5, *F.g*.9 and *F.g*.10 (33%) of the *F. graminearum* isolates were highly similar to one another; they exhibited similarity coefficients of (87%). The genetic relationships among twelve isolates of *F. graminearum* were determined by Jaccard’s coefficient, Table 3. The matrix of similarity values of SRAP profiles ranged from 42 to 96% among all the isolates. The highest genetic similarity (96%) was recorded between *F.g*.1 and *F.g*.2 which obtained from Dirout followed by (88%) between *F.g*.11 and *F.g*.12. Whereas, the lowest genetic similarity (42%) was recorded between *F.g*.5 and *F.g*.9. The three *F. graminearum* groups identified by SRAP analysis did not share the same pathogenicity to wheat, as well as isolates from within region have been shown to vary in pathogenicity. There were biologically significant differences between the groups, with group B isolates being more aggressive towards wheat than groups A and C.

### Isolation and identification of antagonists

*T. harzianum* and *B. subtilis* were isolated from wheat kernels. *T. harzianum* was isolated on PDA medium at 26°C and identified based on morphological characteristics of mycelia and conidiophores as described by Domsch et al*.*, 1980 and Dhingra and Sinclair, 1995. Whereas, *B. subtilis* was isolated on nutrient agar (NA) at 28°C, and identified based on morphological, culture and biochemical activities that summarized in Table 4 (Skinner and Lovelock, 1979; Sneath et al., 1986; Bergey’s Manual of systematic bacteriology, 2001).

### Effect of *T. harzianum* and *B. subtilis* against *F. graminearum in vitro* and greenhouse

Seven isolates of *T. harzianum* and five isolates of *B. subtilis* were obtained from wheat kernels. *In vitro* and greenhouse studies were conducted to evaluate the efficacy of *Trichoderma harzianum* and *Bacillus subtilis* in control of *F. graminearum*. *In vitro* assay was carried out by dual culture technique and the antagonism was measured as reduction in pathogen colony diameter. *T. harzianum* isolates significantly reduced the growth of *F. graminearum* colonies in culture with the inhibition rate of 51%, 49%, 65%, 63%, 60%, 46% and 48% respectively. Whereas, *B. subtilis* isolates exhibited a medium antifungal effect on the mycelium growth of *F. graminearum* with the inhibition rate of 45%, 55%, 46%, 43% and 42% respectively (Table 5).

In order to gain insight into biological control effect *in situ*, *T. harzianum* and *B. subtilis* were applied in greenhouse. Based on their *in vitro* effectiveness isolate (*T.h*.3) of *T. harzianum* and isolate (*B.s.*2) of *B. subtilis* were selected for greenhouse test. Based on the data under greenhouse conditions, *T. harzianum* was more effective than *B. subtilis* in reducing severity of FHB. *T. harzianum* showed highly reduction in head blight severity (55%), while *B. subtilis* reduced head blight severity by (36.7%) compared with the untreated controls, Table 6. The obtained results revealed that *T. harzianum* and *B. subtilis* significantly reduced the percentage of diseased spikelets under greenhouse conditions. Obtained results indicate that the use of microorganisms that antagonize plant pathogens is risk-free when it results in enhancement of resident antagonists.

## Discussion

In the current study, wheat fields in three districts of Assiut governorate were surveyed for the occurrence of FHB. The results of pathogenicity tests indicated significant differences in aggressiveness among isolates of *F. graminearum*; some isolates produced average of 92.50% disease severity, whereas others were much less virulent with average 54.25%. Isolates from Manfalout appeared to be more variable than the isolates from Dirout and Abuteeg. This difference in pathogenicity between *F. graminearum* groups confirms the need for the vigilant monitoring of potentially infected material and selection of suitable plant breeding strategies.

The SRAP-PCR results of this study give a clear evidence for existence of variations within a small geographical area for *F. graminearum*. The present investigation will help in formulating control measures against the pathogen showing high variability. The SRAP marker allowed the identification of genetic variability of twelve isolates of *F. graminearum* where all the twelve isolates from Assiut have a genetic similarity of 52%. The results of SRAP analysis are generally in compatible with the pathogenicity tests of the isolates of *F. graminearum* which indicate significant level of variation among the twelve isolates of *F. graminearum*. Differences in aggressiveness among isolates may due to genetic recombination, mutation, or selection. In *F. graminearum*, there is large genetic variation within a given population, even in samples collected from a small area within a field (McMullen et al., 1997; Mert-Türk et al., 2014). Several studies reported variation in aggressiveness among *F. graminearum* isolates sampled from various parts of the world within a country and even within populations from individual fields (Akinsanmi et al., 2006; Bai and Shaner, 1996; Cumagun and Miedaner, 2003; Miedaner et al., 2010). As pointed out by Miedaner et al. (2000), the high level of genetic variation in aggressiveness and other characteristics suggests that *F. graminearum* isolates possess a high level of genetic plasticity that may threaten resistant host varieties. Continued monitoring of populations is required to detect such events, which might pose a threat to the FHB-resistant varieties being produced in different countries relying on a limited number of resistance genes. The previous Egyptian study was limited in scope of this topic. Therefore, further studies are required to understand the population structure and establish the degree of genetic diversity of *F. graminearum* from different geographic regions in Egypt. Depending on the results of SRAP profiles, *F. graminearum* isolates were divided into three groups A, B and C on the basis of the geographical origin of the isolates. Previous publications have confirmed the same finding (Carter et al., 2000; Carter et al., 2002; O’Donnell et al., 2000). Results in the present study indicate a high genotypic diversity among the *F. graminearum* isolates in Assiut. Although isolates from different regions clustered together, indicating a relatively high level of genetic exchange between different regions, there was also evidence for diversity related to geographic separation. Diversity studies of *F. graminearum* in other countries (Akinsanmi et al., 2006; Zeller et al., 2004) have revealed a similar population structure (Carter et al., 2000). SRAP markers showed high genetic diversity among *G. zeae* isolates. The significant proportion of variance accounted by the variety compared with the geographic origin of isolates suggests that seedborne inoculum may be contributed to the genetic diversity within the *G. zeae.* Because, inoculum migration together with sexual recombination are probably the main factors affecting the genetic diversity of *G. zeae* populations (Fernando et al., 2006). The current study demonstrated that, SRAP analysis identified considerable diversity within *F. graminearum*. Moreover, Dinolfo et al., 2015 demonstrated the efficacy of SRAP molecular marker to amplify open reading frames in fungus.

Biological control is an efficient and environmentally friendly way to reduce the disease severity of FHB. The findings of the present study declared that, all the antagonists inhibited the growth of *F. graminearum* in culture, however *T. harzianum* were the most effective, inhibiting the growth of the pathogen. In greenhouse, *T. harzianum* reduced the percentage of diseased spikelets by 55%, while *B. subtilis* reduced the percentage of diseased spikelets by 36.7%. Trichoderma and Bacillus have the highest inhibitory effect to pathogens in culture (Müllenborn et al., 2007; Perello et al., 2002; Schunmacher et al., 2007). Production of antifungal secondary metabolites by Trichiderma can induce resistance of plants against infection by pathogenic microorganisms. Trichoderma strains exert biocontrol against fungal phytopathogens either indirectly, by competing for nutrients and space, modifying the environmental conditions, or promoting plant growth and plant defensive mechanisms and antibiosis, or directly, by mechanisms such as mycoparasitism (Benítez et al., 2004). Trichoderma strains grow rapidly and they are naturally resistant to many toxic compounds, including herbicides, fungicides and pesticides such as DDT, and phenolic compounds (Chet et al., 1997) and because the strains recover very rapidly after the addition of sublethal doses of some of these compounds. Resistance to toxic compounds may be associated with the presence in Trichoderma strains of ABC transport systems (Harman et al., 2004). Inhibition occurred by *B. subtilis* against *F. graminearum* probably due to the late production of antifungal metabolites or competition for nutrients and space rather than inhibition by antimicrobial secretion (Asak and Shoda, 1996; Agarry et al., 2005; Melo, 1998). *B. subtilis* strains produce a broad spectrum of antimicrobial compounds, including predominantly peptides as well as a couple of non-peptidic compounds such as polyketides, an aminosugar, and a phospholipid (Stein, 2005). The antifungal effects might have been due to one or more antifungal compounds produced by this biocontrol agent. Chitin is a common constituent of fungal cell walls (Cohen-Kupiec and Chet, 1998). *B. subtilis* could produce chitinase on chitin-amended media. It indicates that *B. subtilis* could break down cell wall of *F. graminearum* by producing chitinase. The cell wall of fungi provides both protective and aggressive functions. If removed or weakened, the fungi die unless they are osmotically protected (Latgé, 2007). It may be presumed that growth inhibition of *F. graminearum* by *B. subtilis* strains in our study might be due to the production of antimicrobial compounds or competition for nutrients and space.

In conclusion, the results demonstrate that SRAP technique is a useful marker system in determining the genetic characterization of isolates of *F. graminearum*. This difference in pathogenicity between *F. graminearum* groups confirms the need for the vigilant monitoring of potentially infected material and selection of suitable plant breeding strategies. Biocontrol agents could play an important role in organic cereal production. In conventional production, such agents may extend protection of spikes past the flowering stage when fungicides can no longer be applied. Certain strains of spore producing bacteria (*B. subtilis*) and fungi (*T. harzianum*) have shown promise results for the control of FHB.

## Figures and Tables

**Table 1:** Sequences of the SRAP primers and 16 primer combinations used to amplify *F. graminearum* genomic DNA

No	Primer combination	Forward primer		Reverse primer	
	
		Name	Primer sequence 5′–3′	Name	Primer sequence 5′–3′
1	Em1- Me2	Me2	TGA GTC CAA ACC GGA GC	Em1	GAC TGC GTA CGA ATT AAT
2	Em2- Me3	Me3	TGA GTC CAA ACC GGA AT	Em2	GAC TGC GTA CGA ATT TGC
3	Em2- Me4	Me4	TGA GTC CAA ACC GGA CC	Em2	GAC TGC GTA CGA ATT TGC
4	Em2- Me6	Me6	TGA GTC CAA ACC GGA CA	Em2	GAC TGC GTA CGA ATT TGC
5	Em3- Me3	Me3	TGA GTC CAA ACC GGA AT	Em3	GAC TGC GTA CGA ATT GAC
6	Em3- Me6	Me6	TGA GTC CAA ACC GGA CA	Em3	GAC TGC GTA CGA ATT GAC
7	Em3- Me4	Me4	TGA GTC CAA ACC GGA CC	Em3	GAC TGC GTA CGA ATT GAC
8	Em3- Me10	Me10	TGA GTC CAA ACC GGA AA	Em3	GAC TGC GTA CGA ATT GAC
9	Em5- Me10	Me10	TGA GTC CAA ACC GGA AA	Em5	GAC TGC GTA CGA ATT AAC
10	Em6- Me4	Me4	TGA GTC CAA ACC GGA CC	Em6	GAC TGC GTA CGA ATT GCA
11	Em11- Me3	Me3	TGA GTC CAA ACC GGA AT	Em11	GAC TGC GTA CGA ATT CTA
12	Em11- Me4	Me4	TGA GTC CAA ACC GGA CC	Em11	GAC TGC GTA CGA ATT CTA
13	Em11- Me6	Me6	TGA GTC CAA ACC GGA CA	Em11	GAC TGC GTA CGA ATT CTA
14	Em11- Me8	Me8	TGA GTC CAA ACC GGA CT	Em11	GAC TGC GTA CGA ATT CTA
15	Em14- Me3	Me3	TGA GTC CAA ACC GGA AT	Em14	GAC TGC GTA CGA ATT.CTT
16	Em14- Me4	Me4	TGA GTC CAA ACC GGA CC	Em14	GAC TGC GTA CGA ATT.CTT

**Table 2:** Pathogenicity tests of *F. graminearum* isolates on wheat (Sakha-69) under greenhouse conditions

Isolates No	Geographical origin of isolates	Diseased spikelets (%)*
*F.g.*1	Group A Dirout*-* Assiut	68.00 F
*F.g.*2		62.75 G
*F.g.*3		73.25 E
*F.g.*4		75.50 E
*F.g.*5		79.50 CD

*F.g.*6	Group B Manfalout- Assiut	84.50 B
*F.g.*7		78.25 D
*F.g.*8		92.50 A
*F.g.*9		81.00 C
*F.g.*10		78.50 CD

*F.g.*11	Group C Abuteeg- Assiut	56.50 H
*F.g.*12		54.25 H

Uninfected control		0.0 I

* Means within the same column followed by different letters are significantly different (*P* ≤ 0.05) based on LSD.

**Table 3:** Similarity matrix of *F. graminearum* isolates (Jaccard’s Coefficient)

	*Fg*-1	*Fg*-2	*Fg*-3	*Fg*-4	*Fg*-5	*Fg*-6	*Fg*-7	*Fg*-8	*Fg*-9	*Fg*-10	*Fg*-11	*Fg*-12
*Fg*-1	1											
*Fg*-2	0.963	1										
*Fg*-3	0.893	0.926	1									
*Fg*-4	0.867	0.833	0.774	1								
*Fg*-5	0.758	0.781	0.781	0.875	1							
*Fg*-6	0.462	0.474	0.474	0.475	0.488	1						
*Fg*-7	0.512	0.488	0.488	0.561	0.571	0.667	1					
*Fg*-8	0.462	0.474	0.474	0.513	0.525	0.818	0.667	1				
*Fg*-9	0.514	0.486	0.486	0.487	0.429	0.639	0.778	0.639	1			
*Fg*-10	0.556	0.528	0.528	0.526	0.463	0.595	0.778	0.595	0.871	1		
*Fg*-11	0.594	0.613	0.613	0.606	0.571	0.588	0.595	0.588	0.606	0.559	1	
*Fg*-12	0.588	0.606	0.606	0.647	0.611	0.583	0.59	0.541	0.556	0.556	0.889	1

**Table 4:** Morphological and physiological characteristics of bacterial isolates

	Characteristics	Reaction of isolates: 1, 2, 3, 4 and 5
1	Shape of cell	Rods
2	Motility	+Ve
3	Gram staining	+Ve
4	Endospore production	+Ve
5	Hydrolysis of casein	+Ve
6	Gelatin liquefaction	+Ve
7	Urea test	−Ve
8	Nitrate reduction	+Ve
9	Starch hydrolysis	+Ve
10	Levan production	−Ve
11	Catalase test	+Ve
12	Indole formation	−Ve
13	Esculin hydrolysis	+Ve
14	Anaerobic growth	−Ve
15	Methyl red test	+Ve
16	Oxidase	−Ve
17	Acid from: D- Glucose	+Ve
	L- Arabinose	+Ve
	D- Xylose	+Ve
	D- Mannitol	+Ve
18	Growth at pH:	
	6.8	+Ve
	5.7	+Ve
19	Growth in NaCl: 2.0%	+Ve
	5.0%	+Ve
	7.0%	+Ve
	10.0%	−Ve
20	Growth at: 5.0°C	−Ve
	10°C	+Ve
	30°C	+Ve
	40°C	+Ve
	50°C	−Ve

Legend: −Ve = Negative reaction; +Ve = positive reaction

**Table 5:** Effect of *T. harzianum* and *B. subtilis* on colony diameter of *F. graminearum* in dual culture

Treatments	Antagonism class	Colony diameter of *F. graminearum* (cm)*	Inhibition of *F. graminearum* growth (%)
*F.g*.+*T.h*.1	1.5	4.37 EF	51.38
*F.g*.+*T.h*.2	1.7	4.55 DE	49.44
*F.g*.+*T.h*.3	1.0	3.12 I	65.27
*F.g*.+*T.h*.4	1.5	3.30 HI	63.33
*F.g*.+*T.h*.5	1.2	3.60 GH	60.00
*F.g*.+*T.h*.6	1.7	4.85 BCD	46.11
*F.g*.+*T.h*.7	2.0	4.60 CDE	48.88
*F.g*.+*B.s.*1	-	4.95 BCD	45.00
*F.g*.+*B.s.*2	-	4.05 FG	55.00
*F.g*.+*B.s.*3	-	4.80 BCDE	46.66
*F.g*.+*B.s.*4	-	5.05 BC	43.88
*F.g*.+*B.s.*5	-	5.15 B	42.77
Control	-	9.0 A	0.0

* Means within the same column followed by different letters are significantly different (*P* ≤ 0.05) based on LSD: *F.g*.= *F. graminearum*; *T.h.= T. harzianum*; *B.s.= B. subtilis.*

**Table 6:** Influence of *T. harzianum* and *B. subtilis* on FHB incited by *F. graminearum* on wheat cultivar Sakha-69 under greenhouse conditions

Treatments	Diseased spikelets (%)*	Reduction (%)
*F.g*.+*T.h*.3	41.00 C	55.06
*F.g*.+*B.s.*2	57.75 B	36.71
*F.g*. alone (positive control)	91.25 A	0.0
Uninfected control	0.0 D	-

* Means within the same column followed by different letters are significantly different (*P* ≤ 0.05) based on LSD: *F.g*.= *F. graminearum*; *T.h.= T. harzianum*; *B.s.= B. subtilis.*
